# Preventing Vector-Borne Transmission of Zika Virus Infection During Pregnancy, Puerto Rico, USA, 2016–2017^1^

**DOI:** 10.3201/eid2611.201614

**Published:** 2020-11

**Authors:** Katherine Kortsmit, Beatriz Salvesen von Essen, Lee Warner, Denise V. D’Angelo, Ruben A. Smith, Carrie K. Shapiro-Mendoza, Holly B. Shulman, Wanda Hernández Virella, Aspy Taraporewalla, Leslie Harrison, Sascha Ellington, Wanda D. Barfield, Denise J. Jamieson, Shanna Cox, Karen Pazol, Patricia Garcia Díaz, Beatriz Rios Herrera, Manuel Vargas Bernal

**Affiliations:** Centers for Disease Control and Prevention, Atlanta, Georgia, USA (K. Kortsmit, B. Salvesen von Essen, L. Warner, D.V. D’Angelo, R.A. Smith, C.K. Shapiro-Mendoza, H.B. Shulman, A. Taraporewalla, L. Harrison, S. Ellington, W.D. Barfield, S. Cox, K. Pazol);; Puerto Rico Department of Health, San Juan, Puerto Rico, USA (W. Hernández Virella, P. Garcia Díaz, B. Rios Herrera, M. Vargas Bernal);; Emory University School of Medicine, Atlanta (D.J. Jamieson)

**Keywords:** insect repellent, microcephaly, PRAMS-ZPER, pregnancy, prenatal counseling, prevention strategies, Puerto Rico, vector-borne transmission, viruses, Zika prevalence, Zika virus, United States

## Abstract

We examined pregnant women’s use of personal protective measures to prevent mosquito bites during the 2016–2017 Zika outbreak in Puerto Rico. Healthcare provider counseling on recommended measures was associated with increased use of insect repellent among pregnant women but not with wearing protective clothing.

During 2016–2017, Puerto Rico had active transmission of Zika virus (ZIKV). During January 27, 2016–June 10, 2017, the Puerto Rico Department of Health (PRDH) reported 40,357 confirmed cases of ZIKV infection, including 3,833 cases among pregnant women (*1*–*3*). Because of the severity of adverse birth outcomes (e.g., brain and eye abnormalities, microcephaly, other birth defects) linked to maternal ZIKV infection (*4*,*5*), the Centers for Disease Control and Prevention (CDC) (*6*–*9*) and PRDH (*10*) released guidance for preventing ZIKV. In areas where ZIKV transmission was active, pregnant women were advised to prevent mosquito bites by wearing protective clothing (long-sleeved shirts and long pants), and by using Environmental Protection Agency (EPA)–registered insect repellent (*6*–*10*). 

## The Study

The Pregnancy Risk Assessment Monitoring System–Zika Postpartum Emergency Response Study (PRAMS-ZPER) was conducted in Puerto Rico by PRDH and CDC to assess women’s use of measures to prevent ZIKV infection during pregnancy (*11*). PRAMS-ZPER, a hospital-based survey of women with a recent live birth, was implemented islandwide in 2 phases: August 28–December 3, 2016, and November 1–December 19, 2017. Hospitals with ≥100 births in the year before the study period were eligible to participate. In 2016, all 36 eligible hospitals participated; in 2017, a total of 30 of 31 eligible hospitals with operating maternity wards during the study period participated.

Delivery dates were randomly sampled for each hospital, and delivery logs were used to identify women who delivered on the sampled dates. Eligibility criteria are described elsewhere (*12*,*13*). In 2016, women were approached 24–36 hours after their infants were delivered (*12*). In 2017, because the environment after Hurricane Maria resulted in early hospital discharges, women were approached within 24 hours postdelivery unless hospital staff recommended otherwise (e.g., mothers who had not yet recuperated). Response rates were 80.6% in 2016 and 94.4% in 2017. 

In previous research, we reported 2016 PRAMS-ZPER data on maternal use of ZIKV prevention measures (*12*) and on the association between provider counseling on condom use to prevent ZIKV infection and self-reported use of condoms with sex partners during pregnancy (*13*). In this analysis, we report on the association between counseling from prenatal care providers and women wearing protective clothing and using insect repellent during pregnancy to prevent ZIKV infection using data from 2016 and 2017. We assessed the prevalence of wearing protective clothing and using repellent overall and by select maternal characteristics (e.g., education level, marital status; Table 1). We constructed separate multivariable, survey-weighted, logistic regression models for each study year to examine maternal characteristics associated with receiving provider counseling on wearing protective clothing and using repellent to prevent mosquito bites. We constructed separate multivariable models to examine associations between factors identified a priori, including receiving provider counseling, with wearing protective clothing daily and using repellent frequently (defined as use every day in 2016 and use always in 2017) when outside during pregnancy. Each model was further adjusted for maternal characteristics, health district region, and delivery month. 

**Table 1:** Adjusted weighted prevalence estimates and prevalence ratios of receiving provider counseling on personal protective measures during pregnancy to prevent vector-borne transmission of Zika virus by maternal characteristics, PRAMS-ZPER, Puerto Rico, USA, 2016–2017*

Maternal characteristics	Received provider counseling on types of clothes to wear to prevent mosquito bites						Received provider counseling about using mosquito repellent				
	2016, n = 2,241†			2017, n = 1,375†			2016, n = 2,244†			2017, n = 1,382†	
	%‡	aPR (95% CI)‡		%‡	aPR (95% CI)‡		%‡	aPR (95% CI)‡		%‡	aPR (95% CI)‡
Total§	87.1	NA		79.8	NA		92.0	NA		90.2	NA
Age, y											
≤19	90.0	1.09 (1.02–1.17)¶		82.2	0.97 (0.89–1.06)		91.5	1.00 (0.96–1.05)		95.9	1.03 (0.99–1.08)
20–34	87.3	1.06 (1.00–1.13)		78.7	0.93 (0.88–0.98)¶		92.1	1.01 (0.97–1.05)		88.9	0.96 (0.93–1.00)
≥35	82.4	Referent		84.4	Referent		91.3	Referent		92.6	Referent
Education level											
<HS diploma	86.7	0.99 (0.94–1.06)		77.2	0.97 (0.88–1.06)		93.4	1.02 (0.98–1.05)		84.2	0.93 (0.85–1.01)
HS diploma	86.7	0.99 (0.96–1.03)		80.8	1.01 (0.96–1.07)		92.1	1.00 (0.98–1.03)		89.9	0.99 (0.96–1.03)
>HS	87.2	Referent		79.7	Referent		91.8	Referent		90.6	Referent
Marital status during pregnancy											
Unmarried	86.6	0.99 (0.95–1.02)		79.5	0.99 (0.95–1.04)		92.1	1.00 (0.98–1.03)		90.0	0.99 (0.96–1.02)
Married	87.9	Referent		80.3	Referent		91.8	Referent		90.6	Referent
Prenatal WIC participation											
Yes	88.0	1.11 (1.03–1.19)¶		81.0	1.11 (1.03–1.20)¶		92.3	1.04 (0.99–1.10)		90.7	1.04 (1.00–1.09)
No	79.5	Referent		73.0	Referent		88.7	Referent		87.1	Referent

*On the 2016 PRAMS-ZPER survey, women were asked about receiving counseling from a healthcare provider about using mosquito repellent on their skin only. On the 2017 PRAMS-ZPER survey, the question was expanded to ask about mosquito repellent on their skin or clothing. aPR, adjusted prevalence ratio; HS, high school; NA, not applicable; PRAMS-ZPER, Pregnancy Risk Assessment Monitoring System–Zika Postpartum Emergency Response Study; WIC, Special Supplemental Nutrition Program for Women, Infants, and Children. †Unweighted sample size; sample size varies because of missing responses. ‡Prevalence and prevalence ratio estimates adjusted for maternal age, education, marital status, prenatal WIC participation, infant birth month, and health district region (Aguadilla, Arecibo, Bayamon, Caguas, Fajardo, Mayaguez, Metro, or Ponce). §Unadjusted prevalence estimates.  ¶Statistically significant result.

Among 3,806 combined respondents in 2016–2017, nearly all, 99.4%, received prenatal care. Among those who received prenatal care, 87.8% participated in the Special Supplemental Nutrition Program for Women, Infants, and Children (WIC) during pregnancy, 79.0% were 20–34 years of age, 69.5% had >high school education, and 68.6% were unmarried. 

Most women reported receiving provider counseling during pregnancy to wear protective clothing in 2016 (87.1%) and 2017 (79.8%) (Table 1); however, few women reported wearing protective clothing daily during either year (11.3% in 2016 and 7.9% in 2017) (Table 2). In 2016, the prevalence of wearing protective clothing was lower among women ≤19 years (7.8%; adjusted prevalence ratio [aPR] 0.46, 95% CI 0.27–0.77) and 20–34 years (11.1%; aPR 0.65, 95% CI 0.46–0.94) compared with those ≥35 years (17.0%) of age and higher among women with less than a high school diploma (20.2%; aPR 2.03, 95% CI 1.38–2.99) and a high school diploma (13.2%; aPR 1.33, 95% CI 1.01–1.73) compared with those who had more than a high school education (9.9%). In 2017, the prevalence of wearing protective clothing was lower among women 20–34 years compared with ≥35 years of age (7.1% vs 13.5%; aPR 0.52, 95% CI 0.36–0.76) and higher among women with a high school diploma compared with those with more than a high school education (10.9% vs 7.0%; aPR 1.55, 95% CI 1.13–2.14). Although receiving provider counseling varied by age and WIC participation in 2016–2017 (Table 1), wearing protective clothing did not differ by receiving provider counseling during either study year (Table 2). The most common reason women reported for not wearing protective clothing was that it was too hot (>75%).

**Table 2:** Adjusted weighted prevalence estimates and prevalence ratios of self-reported use of personal protective measures to prevent vector-borne transmission of Zika virus during pregnancy by maternal characteristics and provider counseling, PRAMS-ZPER, Puerto Rico, USA, 2016–2017*

Maternal characteristics	Wore long sleeves and long pants every day						Frequent mosquito repellent use†				
	2016, n = 2,238‡			2017, n = 1,365‡			2016, n = 2,241‡			2017, n = 1,375‡	
	%§	aPR (95% CI)§		%§	aPR (95% CI)§		%§	aPR (95% CI)§		%§	aPR (95% CI)§
Total¶	11.3	NA		7.9	NA		45.4	NA		56.9	NA
Age, y											
≤19	7.8	0.46 (0.27–0.77)#		8.3	0.62 (0.35–1.08)		47.6	0.82 (0.68–0.99)#		53.3	0.87 (0.73–1.04)
20–34	11.1	0.65 (0.46–0.94)#		7.1	0.52 (0.36–0.76)#		43.5	0.75 (0.67–0.85)#		56.6	0.93 (0.84–1.03)
≥35	17.0	Referent		13.5	Referent		57.9	Referent		61.1	Referent
Education level											
<HS diploma	20.2	2.03 (1.38–2.99)#		8.0	1.14 (0.63–2.08)		47.7	1.07 (0.90–1.26)		60.3	1.09 (0.93–1.27)
HS diploma	13.2	1.33 (1.01–1.73)#		10.9	1.55 (1.13–2.14)#		46.4	1.04 (0.93–1.15)		60.6	1.09 (1.00–1.20)
>HS	9.9	Referent		7.0	Referent		44.8	Referent		55.5	Referent
Marital status during pregnancy											
Unmarried	10.8	0.88 (0.68–1.14)		8.6	1.31 (0.92–1.86)		45.6	1.02 (0.92–1.12)		57.4	1.03 (0.95–1.12)
Married	12.3	Referent		6.5	Referent		44.8	Referent		55.8	Referent
Prenatal WIC participation											
Yes	11.2	0.93 (0.63–1.36)		8.1	1.15 (0.73–1.81)		46.6	1.29 (1.07–1.57)#		58.4	1.20 (1.07–1.35)#
No	12.1	Referent		7.0	Referent		36.0	Referent		48.5	Referent
Receipt of provider counseling on types of clothes to wear to prevent mosquito											
Yes	11.3	0.99 (0.73–1.35)		8.2	1.15 (0.79–1.68)		NA	NA		NA	NA
No	11.4	Referent		7.1	Referent		NA	NA		NA	NA
Receipt of provider counseling on using mosquito repellent											
Yes	NA	NA		NA	NA		46.1	1.23 (1.05–1.46)#		58.9	1.52 (1.29–1.78)#
No	NA	NA		NA	NA		37.3	Referent		38.8	Referent

*On the 2016 PRAMS-ZPER survey, women were asked about receiving counseling from a healthcare provider about using mosquito repellent on their skin only. On the 2017 PRAMS-ZPER survey, the question was expanded to ask about mosquito repellent on their skin or clothing. aPR, adjusted prevalence ratio; HS, high school; NA, not applicable; PRAMS-ZPER, Pregnancy Risk Assessment Monitoring System–Zika Postpartum Emergency Response Study; WIC, Special Supplemental Nutrition Program for Women, Infants, and Children. †Defined as every day use in 2016 and always use in 2017.  ‡Unweighted sample size; sample size varies because of missing responses. §Prevalence and prevalence ratio estimates adjusted for maternal age, education, marital status, prenatal WIC participation, receipt of provider counseling, infant birth month, and health district region (Aguadilla, Arecibo, Bayamon, Caguas, Fajardo, Mayaguez, Metro, or Ponce). ¶Unadjusted prevalence estimates.  #Statistically significant result.

Most women also reported receiving provider counseling during pregnancy about repellent use in 2016 (92.0%) and 2017 (90.2%); receiving counseling did not differ by maternal characteristics. Frequent repellent use was reported by 45.4% of women in 2016 and 56.9% in 2017. In 2016, frequent repellent use was lower among women ≤19 years (47.6%; aPR 0.82, 95% CI 0.68–0.99) and 20–34 years (43.5%; aPR 0.75, 95% CI 0.67–0.85) compared with those ≥35 years of age (57.9%). During 2016–2017, frequent repellent use was higher among women receiving WIC (46.6%; aPR 1.29, 95% CI 1.07–1.57 in 2016; 58.4%; aPR 1.20, 95% CI 1.07–1.35 in 2017) compared with those not receiving WIC (36.0% in 2016 and 48.5% in 2017). Women who received provider counseling on using repellent were also more likely to report frequent repellent use compared with women not receiving counseling in both 2016 (46.1% vs. 37.3%; aPR 1.23, 95% CI 1.05–1.46) and 2017 (58.9% vs. 38.8%; aPR 1.52, 95% CI 1.29–1.78). The most common reason women reported for not using repellent was forgetting to apply or reapply it (>50%).

## Conclusions 

Most women reported being counseled by a prenatal healthcare provider during pregnancy on using repellent and wearing protective clothing to prevent ZIKV infection from mosquito bites. Provider counseling about repellent use was associated with a higher prevalence of frequent repellent use. This finding is consistent with our previous analysis of PRAMS-ZPER data, which showed receiving provider counseling was associated with a higher prevalence of condom use to prevent sexual transmission of ZIKV infection during pregnancy (*13*). In contrast, no significant association was found between receiving provider counseling and wearing protective clothing. Efforts to improve use of other risk-reduction strategies to prevent mosquito bites (e.g., repellent use, removal of standing water, screens on windows) may be beneficial, particularly when barriers, such as hot tropical climates, make wearing protective clothing less feasible. In 2017, the questionnaire was modified to include a question about using repellent on clothing in addition to exposed skin. In addition, changes in conditions after Hurricane Maria may have contributed to the increase in reported repellent use. During the ZIKV outbreak, WIC also implemented efforts to provide participants with targeted education on ZIKV prevention strategies and a prevention kit containing condoms, repellent, a bed net, and larvicide (*14*), which may partially explain increased use of repellent among WIC participants in our analysis. We found a significant association between WIC participation and frequent use of repellent but were unable to further assess the frequency or type of prenatal education WIC recipients received regarding repellent use. Of note, during 2016 only, women were asked whether they received a WIC Zika prevention kit; 77% reported receiving a kit, demonstrating the broad reach of WIC services related to ZIKV prevention among this study sample. 

During prenatal care visits, healthcare providers can help prevent ZIKV infection by counseling pregnant women and their partners about risk-reduction strategies. Provider counseling on repellent and condom use were both associated with increased adoption of practices that reduce the risk of ZIKV infection (*13*). Findings from this study can be applied more broadly to the prevention of other vectorborne diseases among pregnant women, such as dengue, chikungunya, and malaria (*7*).
